# Local radicality and survival outcome of pancreatic cancer surgery

**DOI:** 10.1002/ags3.12273

**Published:** 2019-07-01

**Authors:** Willem Niesen, Thomas Hank, Markus Büchler, Oliver Strobel

**Affiliations:** ^1^ Department of General, Visceral and Transplantation Surgery Heidelberg University Hospital Heidelberg Germany

**Keywords:** lymphadenectomy, pancreatic cancer, radicality, resection margin, surgical resection

## Abstract

Pancreatic cancer remains a therapeutic challenge. Surgical resection in combination with systemic chemotherapy is the only option promising long‐term survival and potential cure. However, only about 20% of patients are diagnosed with tumors that are still in a resectable stage. Even after potentially curative resection and modern regimens for adjuvant chemotherapy, the majority of patients develop local and systemic recurrence resulting in median overall survival times of 28‐54 months. The predominance of systemic recurrence and its impact on survival may lead to the assumption that surgical radicality and local control play only minor roles in the treatment of pancreatic cancer. This review provides an overview of the recent literature on surgical radicality and survival outcome in pancreatic cancer. The current evidence on the extent of lymphadenectomy, the prognostic impact of the extent of lymph node involvement, and the impact of the resection margin status on postresection survival are reviewed. Data from recent studies performed in the context of modern surgery and adjuvant therapy provide good evidence of a considerable impact of local radicality on survival after pancreatic cancer surgery. Surgical techniques that have been developed to refine oncological resections and to increase local control as well as resectability are highlighted. These techniques include artery‐first approaches, level‐3 dissection with removal of the periarterial nerve plexus, the triangle operation, and extended resections. Local radicality and quality of surgical resection remain among the most important parameters that determine the chances for survival in patients with non‐metastatic pancreatic cancer.

## 1 INTRODUCTION

The overall prognosis of pancreatic ductal adenocarcinoma (PDAC) remains poor and its therapy remains challenging.^1^ Surgical resection in combination with systemic therapy offers the only chance for long‐term survival or potential cure.^1^ However, only about 20% of patients with pancreatic cancer are diagnosed with tumors in a resectable stage and in spite of significant progress in surgical resection and chemotherapy, most surgical patients develop local and systemic recurrences resulting in median overall survival of 28‐54 months after potentially curative resection and the best available regimens for adjuvant therapy.^1^, ^2^, ^3^, ^4^, ^5^ The recurrence patterns point to problems with both local surgical radicality and early systemic spread with presence of micrometastatic disease at the time of surgical resection as two mechanisms resulting in poor survival outcomes. While the problem of early systemic spread can only be overcome by development of more effective systemic therapies, surgeons may be able to impact on local control by the radicality and quality of surgical resection. However, some surgeons and many oncologists believe that with early systemic recurrence seen in most patients with pancreatic cancer, surgical radicality and local control play only minor roles in this disease.

This review provides an overview of the recent literature on surgical radicality and survival outcome in pancreatic cancer with a focus on the extent of lymphadenectomy and the resection margin status as two surrogate markers of local radicality. Surgical techniques developed to refine oncological resections and to increase local control are highlighted. While strategies of neoadjuvant therapy that are used to achieve resectability in unresectable tumors^6^ may also increase local control in resectable and borderline‐resectable tumors, this review focuses on studies performed in the setting of upfront resection.

## 2 EXTENT OF LYMPHADENECTOMY AND OUTCOME IN PANCREATIC CANCER

Pancreatic cancer is a tumor characterized by early lymphatic invasion and early spread to regional lymph nodes. The rate of lymph node metastases in resectable pancreatic cancer is high at about 70%‐80%.^7^, ^8^, ^9^, ^10^, ^11^, ^12^ The presence of lymph node metastases impacts on tumor stage and is an important prognostic factor associated with decreased survival. In a recent large study performed with a strategy of upfront surgical resection with a radical regional lymphadenectomy and adjuvant chemotherapy, median overall survival in N0 versus N+ tumors was 33.2 months versus 23.6 months and 5‐year survival rates were 31.7% versus 17.4%, respectively.^12^

It has been a long‐standing topic of research and debate among surgeons if the prognosis of pancreatic cancer with lymph node metastases can be improved by extended lymphadenectomy.^13^, ^14^, ^15^, ^16^, ^17^ Five randomized controlled trials published between 1998 and 2014 have compared a “standard” regional versus an “extended” lymphadenectomy for pancreatic head cancer (Table 1).^13^, ^14^, ^15^, ^16^, ^17^ Overall, the individual trials as well as a recent meta‐analysis based on these trials^18^ came to the conclusion that extended lymphadenectomy does not result in improved survival but is associated with increased morbidity and should, therefore, not be recommended as the standard procedure. However, a closer look to the data reveals that although the definitions used for the “standard” and the “extended” lymphadenectomy were quite similar among these trials, there is a considerable heterogeneity and a lack of comparability of data available from these studies, as evidenced by the number of examined lymph nodes. The median numbers of examined lymph nodes are a surrogate marker of the actual extent of lymphadenectomy and vary between 13 and 17 lymph nodes in the “standard” and between 20 and 40 lymph nodes in the “extended” lymphadenectomy groups among the trials. Recent studies from Europe and Japan report median numbers around 23‐26 examined lymph nodes for “regional” lymphadenectomy^4^, ^12^ and, therefore, numbers range between the numbers reported for “standard” and “extended” lymphadenectomy in the available randomized controlled trials on the topic. The Japanese Pancreas Society (JPS) has established a comprehensive nomenclature of the different lymph node stations that are relevant for pancreatic cancer surgery^19^, ^20^ which was not yet consistently used in some of the above‐mentioned randomized trials. This nomenclature has meanwhile been internationally adopted and allowed to set international standards for the extent of lymphadenectomy in pancreatic cancer. Based on this nomenclature the International Study Group on Pancreatic Surgery (ISGPS) released consensus recommendations of a standard regional lymphadenectomy to be performed during pancreatoduodenectomy for pancreatic head cancer and during distal pancreatectomy for pancreatic body and tail cancers in 2014.^21^ Briefly, the principles of a standard lymphadenectomy are defined by a radical removal of all regional lymph nodes including all lymph nodes on the tumor‐oriented side of the celiac axis and the superior mesenteric artery.

**Table 1:** Randomized controlled trials analyzing standard vs extended lymphadenectomy in pancreatic cancer

Study	Years	Patients included	Standard vs extended	Definition of standard and extended	Lymph node retrieval	1/2/3/4/5‐year OS	Median survival	Morbidity	Mortality
Pedrazzoli 1998^13^	1991 ‐ 1994	81	40 standard	Removal of the anterior and posterior pancreaticoduodenal, pyloric, and biliary duct, superior and inferior pancreatic head, and body lymph node stations	Mean: 13.3 median: NA.	All patients: 2 years: 22.2% 3 years: 8.6% 4 years: 7.4%	11 months	NA 47.5%^a^	5%
			41 extended	removal of lymph nodes from the hepatic hilum, along the aorta from the diaphragmatic hiatus to the IMA laterally to both renal hila, with circumferential clearance of the origin of the celiac trunk and SMA	mean: 19.8 median: NA		16.4 months	NA 48.8%^a^	4.9%
					*P* < 0.03		*P* = 0.65	n.s.	n.s.
Yeo 2005^14^	1996 ‐ 2001	294	146 standard	anterior and posterior pancreaticoduodenal lymph nodes (LNS 13, 17) nodes in the lower hepatoduodenal ligament (LNS 12b2, 12c) nodes along the right lateral aspect of the SMA and SMV (some LNS 14b and 14v)	Mean: 17 Median: 16	PDAC only: 3 years: 36% 5 years: 10%	PDAC only: 21 months	NA 33.9%^a^	4%
			148 extended	including LNS 5 and 6, and some 3 and 4, retroperitoneal LN from the right renal hilum to the left border of the aorta, and from the PV to the origin of the IMA including LNS 16a2, 16b1 and LNS 9	Mean: 28.5 Median: 26	PDAC only: 3 years: 38% 5 years: 25%	PDAC only: 20 months	NA 38.7%^a^	2%
					*P* < 0.001	*P* = 0.57	*P* = 0.57	*P* = NA	n.s.
Farnell 2005^15^	1997 ‐ 2003	79	40 standard	including gastric and pyloric nodes (LNS 3, 4, and 6), nodes to the right of the hepatoduodenal ligament (LNS 12b1, 12b2, 12c) anterior/posterior pancreaticoduodenal nodes (LNS 17a, 17b, 13a, 13b) nodes to the right of the SMA (LNS 14a and 14b) nodes anterior to the CHA (LNS 8a)	Mean: 15 Median: 15	3 years: 41% 5 years: 16.4%	26 months	NA 62.5%^a^	0%
			39 extended	removing retroperitoneal soft tissue from the hilum of the kidneys bilaterally and the celiac axis superiorly to the IMA inferiorly circumferential dissection of the CHA (LNS 8p), and the CHA (9), dissection of the hepatoduodenal ligament (LNS 12a1, 12a2, 12p1, 12p2) circumferential dissection of the SMA (LNS 14c, 14d, 14v) para‐aortic lymph nodes from the CA superiorly to the IMA inferiorly (LNS 16)	Mean: 34 Median: 34	3 years: 25% 5 years: 16.5%	18.8 months	NA 34.1%^a^	3%
					*P* < 0.0001	*P* = 0.32	*P* = 0.32	*P* = NA	n.s.
Nimura 2012^16^	2000 ‐ 2003	101	51 standard	anterior and posterior pancreaticoduodenal nodes (LNS 13a, 13b, 17a, 17b) without nerve dissection	Mean: 13.3	3 years: 28% 5 years: 16%	19.9 months	19.6%	0%
			50 extended	lymph nodes around the CHA (LNS 8a, 8p), CA (LNS 9), SMA (Nos. 14p, 14d) and AA between the origin of the CA and the IMA (LNS 16a2, 16b1) dissection of the hepatoduodenal ligament (LNS 12a, 12b, 12p) circumferential nerve dissection around the CHA and SMA semicircumferentially on the right lateral aspect of the CHA	Mean: 40.1	3 years: 18% 5 years: 6%	13.8 months	22%	2%
					*P* < 0.0001	*P* = 0.119	*P* = 0.119	n.s.	n.s.
Jang 2014^17^	2006 ‐ 2009	169	83 standard	lymph nodes around the pancreas head (LNS 13, 17) and gallbladder (LNS 12c) without nerve dissection around the CHA or SMA	Mean: 17.3 Median: NA	2 years: 44.5%	18.8 months	43%	0%
			86 extended	lymph nodes around the CHA (LNS 8), CA (LNS 9), peripancreatic area (LNS 13, 17), hepatoduodenal ligament (LNS 12), SMA (LNS 14), and para‐aortic area (LNS 16) dissection of soft tissue around the hepatoduodenal ligament semicircumferential dissection of the nerve plexus or ganglion on the right side of the CA and SMA	Mean: 33.7 Median: NA	2 years: 35.7	16.5%	32.5%	2.3%
					*P* < 0.001	*P* = 0.401	*P* = 0.401	*P* = 0.16	n.s.

Abbreviations: AA, abdominal aorta; CA, celiac axis; CHA, common hepatic artery; IMA, inferior mesenteric artery; LNS, lymph node station (according to Japanese Pancreas Society); n.s., not significant; NA, not available; PDAC, pancreatic ductal adenocarcinoma; PV, portal vein; SMA, superior mesenteric artery.

^a^According to the meta‐analysis of Dasari.^18^

The term “extended” lymphadenectomy should be reserved for retroperitoneal lymph nodes and such extended lymphadenectomies are not recommended as a standard. Lymph nodes in this area are internationally considered extraregional and, therefore, distant metastases (M1). While some surgeons consider extraregional lymph node metastases to be a contraindication for surgery, small observational studies suggest that the prognosis after their resection is much better than in the setting of distant organ metastases.^22^ In a study focused on the prognostic impact of the extent of lymph node involvement, survival was quite similar after resection of tumors with ≥8 positive regional lymph nodes and resection of additional inter‐aortocaval lymph node metastases (median survival: 18.3 vs 13.6 months; identical 5‐year survival of 9.9% vs 9.9%).^12^ These data suggest that retroperitoneal lymph node metastases are just a more advanced extent of lymph node involvement but do not have the same biological and prognostic implications as distant organ metastases. With a 5‐year survival of around 10%, a significant proportion of patients with inter‐aortocaval lymph node metastases appear to benefit from surgical resection. We, therefore, recommend taking frozen section biopsies of retroperitoneal lymph nodes whenever their involvement is suspected based on imaging or surgical exploration and to perform an extended retroperitoneal lymphadenectomy if metastases are confirmed. A sensible alternative may be to abandon upfront resection and to administer chemotherapy with a neoadjuvant intention in these selected patients.

Recent observational studies performed in the context of a “radical” regional lymphadenectomy have renewed the international discussion on the prognostic significance of the extent of regional lymph node involvement in pancreatic cancer. Based on the JPS nomenclature of lymph nodes and data available in Japan due to a historically more meticulous approach toward lymphadenectomy in pancreatic cancer, two categories for lymph node‐positive tumors dependent on anatomical groups of regional lymph nodes involved had been already used for prognostic staging in Japan.^23^ In contrast, only one category (N1) for all tumors with regional lymph node metastases irrespective of the extent of lymph node involvement was still used in the seventh edition of the TNM staging manual.^24^ Following up on a study from Japan showing that the number of positive lymph nodes was a powerful predictor of prognosis in pancreatic cancer if a thorough lymphadenectomy is performed^25^ a large study performed in 811 patients undergoing pancreatoduodenectomy for pancreatic cancer between 2001 and 2012 demonstrated the possibility to distinguish several prognostic categories of lymph node‐positive cancers dependent on the number of positive lymph nodes (PLN).^12^ In this study median overall survival of patients with regional lymph node metastases ranged from 31.1 months with one PLN to 18.3 months with ≥8 PLN. The differences in 5‐year survival rates were even more pronounced ranging from 31.4% to 9.9% with one and ≥8 PLN, respectively. The extent of lymph node involvement was confirmed as an independent predictor of overall survival by multivariable analyses with a cut‐off at four PLN.^12^ Together with two smaller confirmatory studies^26^, ^27^ and a population‐based study using the Surveillance, Epidemiology, and End Results Program (SEER) database^28^ the accumulating evidence resulted in a revision of lymph node staging in the eighth edition of the WHO TNM staging manual that now distinguishes pN0, pN1 (1‐3 PLN) and pN2 (4 ≥ PLN).^29^, ^30^ Data from the JPS Pancreatic Cancer Registry between 2001‐2007 demonstrate the same effect when distinguishing N0, N1a (1‐3 PLN), and N1b (≥4 PLN) based on the current Japanese Classification of Pancreatic Cancer (fourth edition, 2017).^20^

While the above‐mentioned studies clearly demonstrate the importance of lymphadenectomy for prognostic staging, direct evidence on a therapeutic effect of lymphadenectomy in pancreatic cancer remains limited. An analysis of the SEER database including 7685 patients with stage I and II pancreatic cancer found that retrieval of 20 or more regional lymph nodes was associated with increased survival in node‐negative as well as node‐positive cancers after adjustment for other prognostic factors.^31^ While the improved survival with ≥20 retrieved lymph nodes in node‐negative cancers may be explained by effects of stage migration, the improved survival with ≥20 retrieved nodes in node‐positive cancers points to a possible therapeutic effect of radical lymphadenectomy.^31^ In contrast to overall survival, the extent of lymphadenectomy may more directly affect local recurrence and recurrence‐free survival. However, good evidence on the effect of lymphadenectomy on the pattern of recurrence after resection for pancreatic cancer is lacking. While early systemic recurrence is more relevant in limiting survival in the majority of patients with pancreatic cancer, about 24% of patients are first affected by isolated local recurrence.^2^ A significant proportion of these isolated local recurrences may originate from regional lymph node metastases. In a radiological study of computed tomography scans performed for surveillance in 99 patients after pancreatic cancer resection, 17% of patients developed isolated local recurrence, including six patients with isolated lymph node recurrences and 11 patients with lymph node and additional perivascular recurrences.^32^ In a study on re‐resection for isolated local recurrence of pancreatic cancer, 41 (72%) of 57 patients with isolated local recurrence proven histologically upon surgical exploration, underwent successful re‐resection associated with a median survival of 26 months after re‐resection.^33^ The majority of these recurrences was perivascular or located in locoregional or retroperitoneal lymph nodes.

In conclusion, the current evidence supports a radical locoregional lymphadenectomy as for example recommended by the ISGPS as a minimum standard lymphadenectomy during pancreatic cancer surgery.^21^ Extended retroperitoneal (paracaval/inter‐aortocaval/para‐aortic) lymphadenectomy should not be performed as a standard procedure because it does not improve overall survival but may increase complications if applied as a standard procedure to unselected patients. In contrast, extended lymphadenectomy may be indicated in selected patients with suspected lymph node metastases in this location during upfront resections or after neoadjuvant therapy. In selected patients with isolated lymph node recurrences that occur during surveillance after pancreatic cancer resection, surgical re‐resection can be considered.

Based on the oncological principles of radical *en bloc* tumor resection, the extent of regional lymphadenectomy is closely connected with the extent and local radicality of resection around the major vessels, especially the celiac axis and the superior mesenteric artery, as discussed in the following section. Surgeons who follow the principles and techniques described below will “automatically” perform an adequate regional lymphadenectomy.

## 3 RESECTION MARGIN STATUS AND SURVIVAL IN PANCREATIC CANCER

In addition to the extent of lymphadenectomy, the resection margin status (R‐status) is another important surrogate marker for surgical radicality and another important prognostic factor that can be influenced by surgical quality, strategy, and technique. The R‐status has become a main focus of recent studies on pancreatic cancer surgery. The need for a standardized pathological work‐up not only of the transection margins but especially of the circumferential margins with inking of all margins and axial slicing was first proposed and published by the groups in Leeds and Heidelberg in 2006 and 2008.^34^, ^35^ Inking allowed better identification and assessment of “circumferential” and mobilization margins, especially of the medial and posterior margins toward the superior mesenteric margins that are most frequently involved in pancreatic cancer. In consideration of the discontinuous spread of pancreatic cancer cells at the invasion margins and especially at sites of perineural invasion, the new protocols for pathological work‐up were accompanied by a revised strict definition of the R‐status, requiring a 1‐mm tumor‐free margin between the closest cancer cell and any margin in order to call an R0 status.^34^, ^35^ Both studies showed that based on the new protocols and definitions, the majority of resections for pancreatic cancer were R1 resections, the medial and posterior margins (located toward the superior mesenteric artery and the celiac axis) were most frequently involved.^34^, ^35^ While the need for assessment of circumferential margins was quickly accepted around the world, the strict definition of the R0 status based on the “1‐mm rule” was adopted in Europe but not accepted internationally and has not been commonly used in studies from the USA or Asia.^4^, ^36^, ^37^ This resulted in a considerable lack of comparability of data on the frequencies and the prognostic impact of R0 and R1 resections. The ISGPS reacted in 2014 by releasing consensus definitions for extended pancreatic resections and borderline‐resectable pancreatic cancer that included not only the recommendation to report the resection margin status based on assessment of seven distinct margins but also supported reporting on a 1‐mm free margin.^38^, ^39^ The first comprehensive systematic review and meta‐analysis on the topic included 19 studies with a total of 4376 patients and highlighted the considerable heterogeneity of reported R0 and R1 rates: studies using the 1‐mm rule and assessing at least six margins reported only 29% R0 rates, while studies still applying a 0‐mm rule reported 72% R0 resections.^40^ While the authors of this meta‐analysis clearly demonstrated that resection margin data originating from contexts with different definitions and work‐up are not at all comparable, they were unable to draw valid conclusions as to the prognostic significance of the R‐status due to heterogeneity in reporting of survival.^40^ Two large cohort studies based on the new protocol for margin assessment and the 1‐mm rule clearly established a considerable impact of the resection margin status on overall survival after pancreatoduodenectomy for pancreatic head cancers and after total pancreatectomy or distal pancreatectomy for pancreatic body and tail cancers, respectively.^41^, ^42^ In 561 patients with pancreatoduodenectomies for pancreatic head adenocarcinoma, 112 (20%) had a “true” R0 resection (>1‐mm tumor‐free margin), 123 (22%) patients had R1 (≤1 mm, but no direct margin involvement) status and 326 (58%) patients had R1 with direct margin involvement. The 5‐year overall survival rates associated with R0 (>1 mm), R1 (≤1 mm) and R1 (direct) resections were 37.7%, 30.1%, and 20.3%, respectively.^41^ The 5‐year overall survival rate for the favorable subgroup of “true R0” without lymph node metastases (pN0, R0) was as high as 62.2%.^41^ In a second study on 455 patients, the prognostic impact of the R‐status was confirmed for tumors located in the pancreatic tail and body treated by distal pancreatectomy (n = 218) or total pancreatectomy (n = 237).^42^ R0 (>1 mm) resections were achieved in 23.5% of these resections. Median overall survival times for patients with R0 (1 mm), R1 (≤1 mm) and R1 (direct) status were 62.4, 24.6 and 17.2 months respectively, with 5‐year survival rates of 62.6%, 16.8% and 13.0%.^42^ In both studies, the R‐status was demonstrated to be an independent predictor of survival.^41^, ^42^

Recent clinical trials and observational studies that present data on resection margins and survival are summarized in Table 2.^3^, ^4^, ^41^, ^42^, ^43^, ^44^, ^45^ These studies show that with modern surgery and adjuvant chemotherapy, median survival times are around 40 months after R0 and 25 months after R1 resections and 5‐year survival rates are around 35% and 10%, respectively. Some may hypothesize, that with more effective regimens for adjuvant chemotherapy, surgical resection margin status may lose its prognostic relevance. However, the results of the recent ESPAC4 demonstrate that the R‐status remains relevant even with effective adjuvant chemotherapy: the median overall survival in the group receiving the more effective combination regimen gemcitabine and capecitabine was 39.5 months after R0 versus only 23.7 months after R1 resection.^3^ In a multivariable analysis, R‐status was confirmed as an independent predictor of survival in this study.^3^

**Table 2:** The effect of positive resection margins on survival in pancreatic cancer

Study	Type of study	Patients included	Type of surgery	R‐definition	R0/R1 rate, absolute (%)	Years	Median survival	5‐year survival rate	Adjuvant (chemo‐)therapy
Uesaka 2016^4^ JASPAC 01	RCT	385	257 (68%): PD 116 (13%): DP 4 (19%): TP	0‐mm rule	R0 > 0 mm: 49 (13%) R1 0 mm: 328 (87%)	2007‐2010			Yes: 98.7%
		190 GEM	136 (72%): PD 50 (26%): DP 4 (2%): TP		R0 > 0 mm: 26 (14%) R1 0 mm: 164 (86%)		25.5 months	24.4%	
		187 S‐1	121 (65%): PD 66 (35%): DP 0 (0%): TP		R0 > 0 mm: 23 (12%) R1 0 mm: 164 (88%)		46.5 months	44.1%	
Demir 2017^43^	Retrospective single‐center	254	174 (67.5%): PD 44 (17.3): DP 36 (14.2%): TP	0‐mm rule	R0 > 0 mm: 153 (60.2%) R1 0 mm: 101 (39.8%)	2007‐2014	R0 > 0 mm: 28.6 months R1 0 mm: 16.5 months	All patients: 22.5%	Yes: 92%
							PD: R0 > 0 mm: 31.8 months R1 0 mm: 14.5 months		
				1‐mm rule	R0 > 1 mm: 109 (42.9%) R1 0‐1 mm: 145 (57.1%)		R0 > 1 mm: 31.7 months R1 0‐1 mm: 17.1 months		
							PD: R0 > 1 mm: 41.2 months R1 0‐1 mm: 16.8 months		
Nitta 2017^44^	Retrospective single‐center	117	107 (91%): cPD 10 (9%): ppPD	0‐mm rule	R0 > 0 mm: 95 (81%) R1 0 mm: 22 (19%)	1999‐2010	R0 > 0 mm: 17 months R1 0 mm: 12 months	NA	Yes: 46 (39%) No: 71 (61%)
				1‐mm rule	R0 > 1 mm: 30 (26%) R1 0‐1 mm: 87 (74%)		R0 > 1 mm: 20 months R1 0‐1 mm: 14 months		
Neoptolemos 2017^3^ ESPAC4	RCT	730	251 (34%): ppPD 370 (51%): cPD 60 (8%): DP 49 (7%): TP	1‐mm rule	R0 ≥ 1 mm: 290 (40%) R1 0‐1 mm: 440 (60%)	2008 ‐ 2011			
		366 GEM					R0 ≥ 1 mm: 27.9 months R1 0‐1 mm: 23 months	16.3%	Yes: 100%
		364 GEM/CAP					R0 ≥ 1 mm: 39.5 months R1 0‐1 mm: 23.7 months	28.8%	Yes: 98%
Ocuin 2017^45^	Retrospective single‐center	310	310 (100%): PD	1‐mm rule	R0 > 1 mm: 130 (41.9%) R1 0‐1 mm: 113 (36.5%) R1 0 mm: 67 (21.6%)	2002‐2014	R0 > 1 mm: 36.9 months R1 0‐1 mm: 22.6 months R1 0 mm: 15.4 months	17.7% 9.7% 4.47%	Yes: 181 (58%) No: 67 (20%) Radiochemotherapy: Yes: 62 (20%)
Strobel 2017^41^	Retrospective single‐center	561	561 PD 72 (12.8%): cPD 427 (76.1%): ppPD 62 (11.1%): prPD	1‐mm rule	R0 > 1 mm: 112 (20%) R1 0‐1 mm: 123 (21.9%) R1 0 mm: 326 (58.1%)	2006‐2012	R0 > 1 mm: 41.6 months R1 0‐1 mm: 27.5 months R1 0 mm: 23.4 months	37.7% 30.1% 20.3%	Yes: 438 (78.1%) No: 72 (12.8%) NA: 51 (9.1%)
Hank 2018^42^	Retrospective single‐center	455	218 DP: (47.9%) 237 TP: (52.1%)	1‐mm rule	R0 > 1 mm: 107 (23.5%) R1 0‐1 mm: 104 (22.9%) R1 0 mm: 244 (53.6%)	2006‐2014	R0 > 1 mm: 62.4 months R1 0‐1 mm: 24.6 months R1 0 mm: 17.2 months	52.6% 16.8% 13%	Yes: 81.5% No: 18.5%

Note

Studies were included on open abdominal pancreatic cancer surgery, beginning from 1996 with focus on survival and resection margin data.

Abbreviations: CAP, capecitabine; cPD, classic pancreatoduodenectomy; DP, distal pancreatectomy; GEM, gemcitabine; PD, pancreatoduodenectomy; PDAC, pancreatic ductal adenocarcinoma; ppPD, pylorus‐preserving pancreatoduodenectomy; prPD, pylorus‐resecting pancreatoduodenectomy; TP, total pancreatectomy.

Despite good evidence for the strict R‐definition using a “1‐mm rule”, there is still no agreement on its general use in current guidelines. The National Comprehensive Cancer Network (NCCN) supports the College of American Pathologists (CAP) protocol that now adopted the strict R1 definition and calls a margin positive if there is tumor at or within 1 mm of the resection margin.^46^, ^47^ The JPS also classify R1, microscopic residual disease, as tumor cells at the margin and recommends to report the shortest distance (in mm) of the invasion site to the closest margin.^48^ The German guidelines were also updated concerning the reporting of the resection margin in a consensus statement^49^ adopting the use of the “circumferential margin” as used for rectal cancer (CRM) into the pathology reporting for pancreatic cancer. While the information on the margin based on the 1‐mm rule is maintained, the former “strict” R0 (1‐mm tumor‐free margin) is now called R0 with the addition “CRM‐”, the former R1 with cancer cells within 1 mm to the closest margin but without direct margin involvement is called R0 with the addition “CRM+”, and only a direct margin involvement is called R1.^49^

The notion reflected in the NCCN (USA) guidelines stating that survival benefits from R1 resections may be comparable to definitive chemoradiation without surgery^50^ is based on outdated studies that report median overall survival times of only 12.3 months for R1 resections^51^, ^52^ and are clearly inconsistent with recent data obtained in the context of the current state of the art of surgery and adjuvant chemotherapy (Table 2).^3^, ^4^, ^41^, ^42^, ^43^, ^44^, ^45^ Of note, the 5‐year overall survival rate after R1 resection with direct margin involvement can still be as high as 20%‐25% and is, therefore, much better than frequently discussed. While obtaining R0 margins is the main goal of every resection performed for pancreatic cancer, R1 resections (even those with direct margin involvement) are still associated with acceptable outcome and should not be interpreted as failure of surgical therapy.

## 4 SURGICAL TECHNIQUES TO INCREASE LOCAL RADICALITY IN PANCREATIC CANCER

In recent years surgical techniques were significantly refined and, together with advances in systemic chemotherapy regimens, resulted in improved outcomes in pancreatic cancer surgery.^1^ These advances have led to the possibility to extend the indications for surgical resection from clearly resectable to locally advanced, borderline‐resectable and previously unresectable tumors. In the following, we want to highlight selected surgical techniques and strategies that contribute to improved local radicality and improved outcomes in pancreatic cancer surgery.

Consistent with the observation that most R1 resections for pancreatic cancer are located at the posterior and medial margins oriented toward the superior mesenteric vessels and the celiac axis,^34^, ^35^ the techniques aiming to increase local radicality are centered on clearance of these vessels as an important and early step during pancreatic cancer resections. With this aim, different techniques have been developed that are today summarized as **“artery‐first approaches”**.^53^ One such technique, the mesenteric approach developed by Nakao et al was already described in 1993 based on a study in 114 patients.^54^ Addressing the superior mesenteric artery (SMA) early and even before mobilization of the pancreatic head, was new and contrary to traditional approaches in pancreatic cancer surgery. A detailed review of the mesenteric approach highlights its advantages for locally advanced tumors with potential SMA involvement and tumors located in the uncinate process.^55^ Hirono et al performed a comparative study in 237 patients undergoing the mesenteric approach (n = 72) and the conventional approach (n = 165) during pancreatoduodenectomy for pancreatic cancer and found the mesenteric approach to be associated with a reduced blood loss (in resectable and borderline‐resectable cancers), an increased R0 rate and better overall survival (in resectable but not in borderline‐resectable cancers).^56^

Over time several other artery‐first approaches were described that may have different advantages dependent on surgical anatomy with the exact location of the tumor and its relation to the vessels.^57^, ^58^, ^59^, ^60^ These different artery‐first approaches were nicely summarized in a technical review published in 2012^53^ and have the following common advantages over traditional techniques: (a) assessment of the resectability of tumors with potential arterial infiltration (which is still considered a contraindication for resection by most surgeons) early during surgical exploration before a point of no return is passed, thus helping to avoid R2 resections; (b) increased radicality at the vessels with the potential to increase the rates of R0 resections; and (c) good control of the vessels resulting in lower blood loss, increased safety resulting in the potential to reduce surgical and overall morbidity of pancreatic resections.

It should be noted that at present the available evidence for these advantages of artery‐first approaches is relatively low because it is limited to retrospective cohort studies.^56^, ^61^, ^62^ A recent systematic review and meta‐analysis of artery‐first versus standard pancreatoduodenectomy identified 16 retrospective cohort or case‐control studies and one very small randomized controlled trial (six vs six patients) on this topic.^63^ In the meta‐analysis of 771 artery‐first versus 701 standard pancreatoduodenectomies the intraoperative blood loss, the need for blood transfusion, the perioperative morbidity, and the rate of clinically relevant postoperative pancreatic fistula were significantly lower, while the R0 rate and overall survival were significantly higher in the artery‐first group.^63^ While these results appear promising, the nature of the included studies suggests a high risk of bias. Studies such as the multicenter randomized controlled MAPLE‐PD trial that is currently being conducted in Japan and compares the mesenteric approach versus conventional pancreaticoduodenectomy in over 350 patients with pancreatic ductal adenocarcinoma^64^ are needed to create high‐level evidence on this important topic and their results are eagerly awaited.

In order to be effective in increasing R0 rates and radicality, the level of dissection at the arteries should be directly at the adventitial layer of the vessels, resulting in a complete dissection of the nerve plexus (and of lymphatic tissue along with it) corresponding to a **level‐3 dissection** according to Inoue et al.^65^ The result of such radical resections at the arteries has also been described as a complete mesopancreas excision.^66^ The rationale to always perform a level‐3 dissection at the SMA and celiac axis at least semicircumferentially, is based on the assumption that with this technique the tumor‐free margin is maximized and tumor cells spreading beyond the tumors along the perivascular nerves (perineural infiltration) are removed. For tumors with contact of more than 180° of contact to the arteries, and especially for resection after neoadjuvant therapy for primarily unresectable tumors, it is frequently necessary to perform a circumferential level‐3 dissection around the SMA and the celiac axis. As these vessels then form a triangle together with the portomesenteric venous axis, this radical resection technique has recently been named the “TRIANGLE operation”.^67^

Indications for surgical resection have been extended toward locally advanced, borderline‐resectable and previously unresectable tumors.^1^ These more advanced tumors can be removed by extended resections that include additional organ and vascular resections.^38^ Such extended resections are increasingly performed around the world in either the upfront setting or after neoadjuvant therapy. Evidence for the best therapy sequencing in borderline‐resectable cancer is still lacking and randomized controlled trials comparing the strategies of upfront resection and adjuvant therapy versus resection after neoadjuvant therapy based on an intention‐to‐treat analysis are urgently needed.^1^ Several large observational studies and meta‐analyses show that survival after extended resections is shorter than after standard resections, owing to the more advanced tumors for which extended resections need to be applied. However, the reported survival after extended resections is still much better than without resection and morbidity and mortality after additional organ resection and venous resections are acceptable. A large meta‐analysis published in 2012 included 19 non‐randomized studies with 2247 patients who underwent potentially curative pancreatectomy.^68^ In 661 (29.4%) patients an extended resection with combined SMV/PV resection was performed. The estimated 1‐, 3‐ and 5‐year overall survival rates for patients undergoing venous resections were 61.3%, 19.4%, and 12.3% compared to 61.8%, 26.6%, and 17% in patients without vascular resection. While estimated blood loss was higher and operation time was longer in the group with venous resection, reported overall morbidity and mortality were similar in both groups.^68^ More recently an observational multicenter study conducted in seven centers in Japan included 937 patients undergoing pancreatoduodenectomy.^69^ Venous resections were frequently performed with almost half (46.4%, n = 435) of all patients undergoing a SMV/PV resection. In this multicenter setting, venous resections did neither increase overall morbidity nor mortality. The median survival for venous resections was 18.5 months versus 25.8 months in smaller tumors without the need for venous resection.^69^ The largest single‐center study on extended resections published in 2016 analyzed 1635 patients with pancreatic cancer, including 611 patients who underwent extended pancreatectomies for advanced tumors.^70^ In this study, median survival after extended resections was 16.1 months and the 5‐year overall survival rate was 11%. Extended total pancreatectomies, but not vascular resections were associated with an increased risk of mortality.^70^

In contrast to venous resections, arterial resections should not be considered for upfront surgery, but for neoadjuvant treatment, which may result in resectability without arterial resections in the majority of cases.^6^, ^67^ An exception may be tumors invading the celiac axis resectable by a distal pancreatectomy with celiac axis resection (DP‐CAR). This procedure relies on arterial blood supply of liver and stomach by collateralization from the SMA via the gastroduodenal artery after resection of the celiac axis without reconstruction. Feasibility and acceptable safety with mortality rates of 3%‐8% after DP‐CAR were demonstrated in several single‐center observational studies.^71^, ^72^ In a recent retrospective international multicenter study including 191 patients undergoing DP‐CAR, the 90‐day mortality rate was 5.5% at five high‐volume but as high as 18% at 18 low‐volume DP‐CAR centers, demonstrating the importance of experience with this rare and complex procedure.^73^ In a multicenter study performed in 20 European centers in 12 countries, the median survival of 68 patients undergoing DP‐CAR was 18 months.^74^ A recent single‐center study reported a very favorable median survival of 38.6 months with a strategy of neoadjuvant therapy followed by DP‐CAR in pancreatic cancer with celiac axis involvement, recommending a neoadjuvant strategy in these patients.^71^ The literature on pancreatic cancer resections with arterial resections apart from DP‐CAR is restricted to case reports and small series with high risk of bias. A meta‐analysis on this topic reported a five‐fold increased risk of mortality after arterial resections versus standard resections and poor 1‐and 3‐year survival rates.^75^ However, some patients may benefit from arterial resections, as long‐term survival can be observed.^75^

Overall, extended resections are associated with shorter survival and may be associated with higher morbidity if compared to standard resections. While venous resections appear to be safe, the need for extended total pancreatectomy and arterial resections are associated with increased morbidity and mortality. Careful patient selection, evaluation of a neoadjuvant strategy and treatment in specialized units are warranted if the need for an extended resection is anticipated. If arterial involvement is anticipated, a strategy of neoadjuvant therapy should usually be preferred.

## 5 CONCLUSION AND PERSPECTIVE

Pancreatic cancer surgery has rapidly evolved in the last decades and along with advances in adjuvant and neoadjuvant therapy, resection rates and survival outcome have significantly improved. However, the prognosis of pancreatic cancer remains poor and most patients eventually develop and die from systemic progression. Therefore, pancreatic cancer has to be considered a systemic disease even in an early clinical tumor stage. In spite of this problem, radical resection with adequate regional lymphadenectomy and radical resection around the large peri‐pancreatic vessels is an important prerequisite for good oncological outcomes. There is ample evidence from recent studies performed in the context of high‐quality radical surgery and modern adjuvant therapy that local radicality, defined by lymph node variables and by resection margin status data, has a profound impact on survival. In modern pancreatic surgery, radical resections can be facilitated and achieved by several techniques, including artery‐first approaches, a level‐3 dissection around the arteries, the TRIANGLE operation, and extended resections with resection of additional organs or vessels. Along with surgical radicality, systemic chemotherapy is the second critical cornerstone of long‐term survival after pancreatic cancer resection. Currently, the best therapy sequencing of surgery and chemotherapy is one of the most important topics in the field of pancreatic cancer surgery and the results of several randomized controlled trials on the strategies of neoadjuvant therapy or upfront resection in resectable and borderline‐resectable pancreatic cancer are eagerly awaited. Independent of the results of these trials, local radicality and quality of surgical resection will remain among the most important parameters determining the chances for survival in patients with non‐metastatic pancreatic cancer.

## CONFLICT OF INTEREST

The authors declare no conflict of interests for this article.
